# Development and Characterization of *Mesembryanthemum crystallinum* L. Extract-Loaded Phytosomes for Enhanced Delivery of Antioxidant Compounds

**DOI:** 10.3390/life16040557

**Published:** 2026-03-29

**Authors:** Irina Fernandes, Ana Iglesias-Mejuto, João M. P. Coelho, Rosa Direito, Catarina P. Reis

**Affiliations:** 1 Faculty of Pharmacy, Universidade de Lisboa, Av. Professor Gama Pinto, 1649-003 Lisboa, Portugal; ijfernandes@edu.ulisboa.pt; 2 Research Institute for Medicines, iMed.ULisboa, Faculty of Pharmacy, Universidade de Lisboa, Av. Professor Gama Pinto, 1649-003 Lisboa, Portugal; ana.mejuto@ff.ulisboa.pt (A.I.-M.); jmcoelho@ciencias.ulisboa.pt (J.M.P.C.); 3 AerogelsLab, I + D Farma Group (GI-1645), Department of Pharmacology, Pharmacy and Pharmaceutical Technology, Faculty of Pharmacy, iMATUS and Health Research Institute of Santiago de Compostela (IDIS), Universidade de Santiago de Compostela, E-15782 Santiago de Compostela, Spain; 4 Instituto de Biofísica e Engenharia Biomédica (IBEB), Faculdade de Ciências, Universidade de Lisboa, 1749-016 Lisboa, Portugal

**Keywords:** *M. crystallinum*, ice plant, phytosomes, antioxidant activity

## Abstract

*M. crystallinum* is an edible halophytic succulent plant rich in phenolic compounds with potential pharmaceutical applications. However, it is known that these phytocompounds generally present low absorption, which hinders their direct use in formulations. Therefore, delivery systems, such as phytosomes, can be regarded as a potential strategy to overcome this disadvantage. This study aimed, for the first time, to prepare extracts from the ice plant using different solvents and to incorporate them into phytosomes. Physicochemical characterization of these phytosomes, their antioxidant activity, as well as the quantification and in vitro release profile of their phenolic and flavonoid compounds were studied. Different extraction solvents were assayed, and Ethanol:Acetone (80:20) achieved a strong antioxidant activity (reaching ca. 71.16%), extracting 3200.3 mg of GAE/100 g and 761.7 mg of QE/100 g of phenolic and flavonoid compounds, respectively. The phytosomal formulation exhibited a mean particle size of 233.80 nm, a polydispersity index of 0.23, and a zeta potential of −27.27 mV. Furthermore, a high encapsulation efficiency (96.63%) of the extracts in the phytosomes was obtained. The in vitro release test indicated that the antioxidant activity was retained, reaching a maximum of 42%, accompanied by a release of 51% of the flavonoid content at the end of the 3 h assay, under the experimental conditions. These findings highlight the potential of phytosomes formulated with *Mesembryanthemum crystallinum* extract as a delivery system for antioxidant phytochemicals.

## 1. Introduction

Rapid economic and technological advancement has brought significant benefits to society, but also contributed to the emergence of numerous lifestyle-related disorders, including diabetes, hypercholesterolemia, and hypertension, which is a major risk factor for cardiovascular disease [1,2]. Moreover, the global population aging and the prevalence of degenerative disorders continue to increase [3]. Neurological conditions, for instance, affect nearly 15% of the population, showing an upward trend along with a consistent decrease in the quality of life [1] and an increasing economic burden on society [1].

As aging progresses, the accumulation of cellular damage contributes to the rise in several health problems [4]. Among these, oxidative stress is a key factor in the pathogenesis of several diseases, namely, diabetes, cancer, neurological diseases, hypertension, and atherosclerosis. Oxidative stress may arise from exogenous sources, such as ultraviolet (UV) radiation or from endogenous processes at the cellular level [5]. It results in intracellular accumulation of reactive oxygen species (ROS) and reactive nitrogen species (RNS) free radicals [6]. An imbalance between ROS production and antioxidants occurs when endogenous neutralization mechanisms are weak [7]. Excess reactive species can damage lipids, proteins, and DNA, leading to cellular damage and inflammation [7,8]. Therefore, there is a clear need to explore alternative and effective therapeutic options to mitigate oxidative stress at the molecular level.

**Figure 1:**
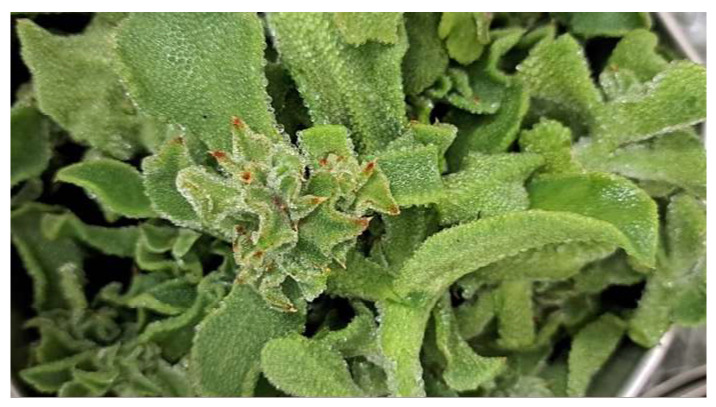
Macroscopic aspect of *Mesembryanthemum crystallinum* L.

*Mesembryanthemum crystallinum* L., commonly known as the ice plant (Figure 1), a reference to the appearance of ice crystals on the surface of leaves and stems, belongs to the Aizoaceae family [9]. This halophyte, native to desert areas in southwestern Africa, and subspontaneous in regions such as the Mediterranean, Australia, and South America, thrives in arid and saline environments [10,11]. Its ability to attract and retain atmospheric moisture throughout the day enables survival under extreme conditions [12]. The ice plant is employed in traditional medicine to treat a myriad of conditions (pneumonia, swelling, skin irritations, or dysentery, among others), and it is still considered an underexplored source of potential therapeutic compounds [9,13].

This plant is known primarily for its antioxidant, anti-inflammatory, antimicrobial [1,14], and potentially antihypertensive and antihyperglycemic properties [12,15,16]. These properties are attributed to its richness in minerals and phytochemicals, such as flavonoids and phenolic acids [12,17,18]. These compounds are primarily localized in specific plant tissues, namely the epithelial bladder cells [10] and the stem surfaces [19,20,21]. Unlike other halophytes, studies identifying and quantifying polyphenolic compounds in *M. crystallinum* L. are still scarce. Variability in reported compositions can be attributed to the fact that the phytochemical concentration in the plant depends on the harshness of the environment where it grows, the season of harvest, and the chosen extraction method [10,22]. Nonetheless, studies on the identification and quantification of the therapeutic compounds of phenolic origin in the ice plant can be found [10,19,20,23], reporting its mineral and phytochemicals richness, such as gallocatechin [24], quercetin [25], p-coumaric acid [26], ferulic acid [27], 4-hydroxybenzoic acid [28], luteolin [10], and diosmin [29], which occur as a response to the extreme environments the plants grow in [17,18]. The phytochemicals present in the plant provide high antioxidant activity against oxidative stress, which is attributed to bioactivities against different pathologies such as hypertension, cancer, or Diabetes Mellitus type 2, among others [30]. The number of hydroxyl groups present in the phytochemicals will determine the strength of the antioxidant activity, by acting as hydrogen and electron donors to stabilize the abovementioned free radicals [31].

However, because of their polarity, low water solubility, and high molecular weight [26,27,28], the application of the natural products present in the ice plant may be limited due to poor absorption rate and oral bioavailability [32]. Furthermore, the complete absorption of polyphenols becomes uncertain once ingested because they complex with other components along the gastrointestinal tract and liver [33]. Throughout this process, polyphenols are set free via the mechanical act of chewing and gastric movement, conjugated, released from conjugations through the different pH digestive environments, and metabolized [34]. All these steps modify the absorption and availability of phytochemicals, depending on their structure. Finally, the phytochemicals are also affected by the compounds they are ingested with, as fibers, alcoholic beverages, and high-fat foods might complex with these phytochemicals, diminishing even more their bioavailability [34].

The low bioavailability of the secondary plant metabolites can be overcome with their formulation within delivery systems [26,27]. With this approach it is possible to maintain the ability to induce a biological response even at a reduced dose, through a precise control over solubility, pharmacokinetic properties, targets, compliance, and toxicity [35]. Phytosomes^®^ (Indena, Milan, Italy) are one of these delivery systems capable of combining water-soluble herbal extracts and poorly soluble compounds with enveloping phospholipids, such as phosphatidylcholine [33]. Phytochemicals with an active hydrogen atom form a hydrogen bond with the hydrophilic choline group [36]; the additional two chains of fatty acids do not participate in forming the complex [31], but they move to create a hydrophobic surface [36]. The stabilization of the phytocompound via hydrogen bonds to the polar head differentiates phytosomes from liposomes, where the actives are distributed in a medium contained by the lipid bilayer [36].

The phytosomal system offers several advantages, including notable stability, enhanced permeability through the biological membranes, and the hepatoprotection offered by the phosphatidylcholine, an amphiphile carrier [32]. They exhibited improved pharmacokinetic and pharmacological properties [27,31], and there are commercialized formulations containing phytochemicals such as *Silybum marianum* (milk thistle) and Oleaselect^®^ (an olive oil-based formulation from the fruit of *Olea europaea* L.), both of which have enhanced antioxidant and cardiovascular attributes [37]. As another example, curcumin-based formulations for metabolic disorders have been shown to mitigate complications in microangiopathy and retinopathy, demonstrating the absorption and accumulation of phytosomes at target sites, the principal advantage regarding the phytocompound alone [32]. Other examples are currently being researched in clinical trials towards their application in cancer, wound healing, and central nervous system ailments [26,31].

Considering the reported health benefits of *Mesembryanthemum crystallinum* and the potential advantages of phytosomal delivery systems, the present study aims to prepare, for the first time, a phenolic-rich extract of the ice plant; characterize its phytochemical composition; and subsequently formulate it into phytosomes. The evaluation of the extract-loaded phytosomes was performed in terms of their physicochemical properties, encapsulation efficiency (EE), phenolic and flavonoid compounds and antioxidant activity.

## 2. Materials and Methods

### 2.1. Materials

Acetone was purchased from Labchem Laborspirit (Lisbon, Portugal), methanol was purchased from Honeywell Riedel-de Haën (Seelze, Germany), and ethanol was purchased from VWR Chemicals (Leuven, Belgium). Quercetin, gallic acid, hydrochloric acid, DPPH, and phosphatidylcholine were acquired from Sigma-Aldrich (St. Louis, MO, USA). Potassium phosphate dibasic was purchased from Honeywell (Offenbach, Germany), and sodium hydroxide was purchased from Fisher Scientific (Loughborough, UK). Sodium carbonate, sodium nitrite, acetic acid and Folin–Ciocalteu reagent were acquired from PanReac (Barcelona, Spain), and aluminum chloride was acquired from Chem-Lab NV (Zedelgem, Belgium). All chemical reagents used were of analytical grade.

### 2.2. Plant Material

The plant *M. crystallinum* L. (Figure 1) was acquired from RiaFresh^®^ (Faro, Portugal) and harvested from Ria Formosa Natural Park with Global/G.A.P. certification. The plant was stored at temperatures between 3 and 7 °C for a maximum of 15 days, until further extraction.

### 2.3. Methods

#### 2.3.1. Acquisition of Extracts

The method used to obtain the phenolic compounds from the ice plant was carried out following a previously reported protocol [38], with some modifications. Five extracts were prepared using 4 different solvents, namely, water, Ethanol:Water 80:20 (*v*/*v*), Ethanol:Water 50:50 (*v*/*v*) and Ethanol:Acetone 80:20 (*v*/*v*). The different extracts were compared to determine the solvents that extract the highest number of compounds from the ice plant. All plant material, before being used, was macerated with the aid of a hand blender. For the water solvent, 100 g of macerated plant was added to 1 L of distilled water and the aqueous extraction was then performed by autoclaving at 121 °C for 15 min to promote efficient extraction of water-soluble phytochemicals under pressurized conditions. After cooling, 10 mL of the aqueous extract was retrieved, and 40 mL of absolute ethanol was added for the removal of polysaccharides. The remaining cooled extract was also kept for further study.

High-temperature treatment facilitates plant cell wall disruption, enhances solvent penetration, and increases mass transfer, thereby improving the recovery of phenolic compounds. In addition, thermal processing contributes to enzyme inactivation (e.g., polyphenol oxidases), which may otherwise lead to degradation of phenolic constituents during extraction. Similar heat-assisted aqueous extraction approaches have been reported for plant matrices to maximize the extraction yield of polar bioactive compounds.

For ethanolic solvents, the ultrasonic extraction method was used. A sample of the plant (25 g) was crushed with a hand blender, and 25 mL of the solvent was added. The plant mixtures were vortexed (Vortex 2000, Heidolph Reax, Schwabach, Germany) and left in an ultrasonic water bath (Vevor, Shanghai, China) for 3 h at room temperature (RT). Aqueous extracts without polysaccharides and ethanolic extracts were centrifuged at 12,000× *g* for 15 min (Centrifuge Z 32 HK, Wehingen, Germany), and the supernatant was collected and afterwards vacuum filtered. The filtered samples were evaporated almost to dryness in an Evaporator R-210 (Buchi, Meierseggstrasse, Switzerland) and were reconstituted with their respective solvents. A portion of the aqueous extract with polysaccharides was also filtered to be used alongside the other extracts. The 5 extracts were stored in Falcon tubes at −20 °C until further analysis, and each extract formulation was obtained in triplicate (n = 3).

#### 2.3.2. Determination of Total Polyphenolic Content of the Extracts

The total polyphenolic compounds were quantified by the Folin–Ciocalteu (FC) method, which measured only the total reducing capacity and may overestimate true polyphenolic content. A method reported by Son et al. [39] was used with some modifications [38]. Firstly, a mixture of 135 µL of distilled water, 750 µL of FC reagent diluted 1:10 *v*/*v*, and 50 µL of extract was vortexed (Vortex 2000, Heidolph Reax, Schwabach, Germany) for 10 s and incubated for 3 min at RT. Then, 600 µL of Na_2_CO_3_ (7.5% *m*/*v*) was added, and the volume was completed to 5 mL with distilled water. The mixture was vortexed (Vortex 2000, Heidolph Reax, Schwabach, Germany) and incubated for 1 h in the dark at RT. The absorbance was then read at 765 nm in the Hitachi UV–visible spectrophotometer U-2000 (Hitachi High-Tech, Tokyo, Japan). Gallic acid solutions were prepared at concentrations of 5–750 mg/L to obtain a calibration curve. The results were expressed in milligrams of gallic acid equivalents per 100 g of fresh ice plant (mg GAE/100 g FW). The experiment was performed in triplicate (n = 3).

#### 2.3.3. Determination of Total Flavonoid Compounds of the Extracts

The quantification of flavonoid compounds was performed by following the method of Son et al. with some modifications [32,33]. A mixture of 1.25 mL of distilled water, 75 µL of NaNO_2_ (5% *m*/*v*) and 250 µL of extract was incubated for 5 min. Then, 150 µL of AlCl_3_ (10% *m*/*v*), 500 µL of NaOH (1 M) and 275 µL of distilled water were added at RT. The absorbance was measured at 510 nm in the Hitachi UV–visible spectrophotometer U-2000 (Hitachi High-Tech, Tokyo, Japan). Quercetin solutions were prepared at concentrations of 8–4000 mg/L to obtain a calibration curve. The results were expressed in milligrams of quercetin equivalents per 100 g of fresh ice plant (mg QE/100 g FW). The experiment was performed in triplicate (n = 3).

#### 2.3.4. Phytosomes Preparation

Phytosomes preparation was carried out according to Direito et al. [38] with some modifications. A 1:1 ratio of the extract and phosphatidylcholine was added to 20 mL of ethanol. After magnetic stirring at 300 rpm for 2 h, 40 mL of acetic acid (2%, *v*/*v*) was added. The organic solvent was then evaporated. The formulation was diluted 1:10 (*v*/*v*) with distilled water and centrifuged (Centrifuge Z 32 HK, Wehingen, Germany) at 12,000× *g* for 20 min. The supernatant was recovered for the determination of the EE, and phytosomes were stored at 4 °C until further analysis.

#### 2.3.5. Physicochemical Characterization of Phytosomes

Phytosomes were diluted in Milli-Q water (1:10, *v*/*v*) for mean particle size and polydispersity index (PdI) analysis and in PBS pH 7.4 (1:10, *v*/*v*) to determine surface charge. A Zetasizer Nano S (Malvern Instruments, Malvern, UK) was used to analyze mean size and PdI by Dynamic Light Scattering (DLS), and a Zetasizer Nano Z (Malvern Instruments, Malvern, UK) was employed to evaluate the surface charge by determining the zeta potential by electrophoretic mobility assay. All measurements were performed in triplicate (n = 3).

#### 2.3.6. Evaluation of EE

The EE was determined using the Folin–Ciocalteu method mentioned above. The mean total phenolic compounds (mg GAE/L) found in the phytosomal formulation supernatant (b) were compared to the mean phenolic compounds (mg GAE/L) found in the free extract (a). The experiment was performed in triplicate (n = 3). The results were expressed as EE (%), following Equation (1):

(1) $$\mathrm{EE}\;(\%)\;=\;\left(\frac{a\;-\;b\;}{a\;}\right)\;\times \;100$$

#### 2.3.7. Quantification of Antioxidant Activity

The antioxidant activity was determined through the 2,2-diphenyl-1-picrylhydrazyl (DPPH) sequestration capacity assay according to the method of Baliyan et al. [40] with some modifications [38]. A total of 24 milligrams of DPPH was dissolved in 100 mL of methanol and filtered. The absorbance of this solution at 517 nm must be above 0.9. Then, 100 µL of the extract was added to 3 mL of the prepared DPPH solution, vortexed (Vortex 2000, Heidolph Reax, Schwabach, Germany) and incubated for 30 min at RT in the dark. The positive control employed was quercetin (10 mg/mL in Milli-Q water), and the negative control was absolute methanol. The absorbance was measured at 517 nm in the Hitachi UV–visible spectrophotometer U-2000 (Hitachi High-Tech, Tokyo, Japan). The experiment was performed in triplicate (n = 3). The results were expressed as antioxidant activity (%), following Equation (2):

(2) $$\mathrm{Antioxidant}\;\mathrm{Activity}\;(\%)\;=\;\left(\frac{\mathrm{Negative}\;\mathrm{Control}\;\mathrm{Abs}\;-\;\mathrm{Sample}\;\mathrm{Abs}\;}{\mathrm{Negative}\;\mathrm{Control}\;\mathrm{Abs}}\right)\;\times \;100$$

#### 2.3.8. In Vitro Release Assay in a Simplified pH-Dependent Model

The release test was performed following the method described by Direito et al. [38]. Firstly, 1 mL of phytosomal formulation was added to 10 mL of HCl/KCl buffer solution, with a pH of 1.2 (USP XXVIII). The mixture was magnetically stirred at RT and 100 rpm for 2 h to simulate the gastrointestinal tract (GIT). The retrieval of constant volumes at pre-determined time intervals was followed by the addition of the same volume of buffer to the mixture, to maintain the initial volume. Afterwards, the mixture was centrifuged (Centrifuge Z 32 HK, Wehingen, Germany) at 12,500× *g* for 10 min. The recovered precipitate was added to 10 mL of phosphate-buffered solution, with a pH of 6.8 (USP XXVIII) and magnetically stirred at 100 rpm for 1 h. Constant volumes were retrieved at pre-determined time intervals, and the same volume of buffer was added to the mixture to maintain the initial volume. All measurements were performed in triplicate (n = 3).

To quantify the phytochemical release, the Folin–Ciocalteu technique, the method for quantification of the flavonoids and the DPPH method were carried out on retrieved samples. The cumulative release was calculated using the following Equations (3) and (4), from the work of Direito et al. [38]:

(3) $$\mathrm{Released}\;\mathrm{Polyphenols}\;(\%)\;=\frac{\sum_{i=1}^{t}\mathrm{Ci}\;\times \;V\;+\;\mathrm{Ct}\;\times \left(V-\sum \mathrm{Vi}\right)}{M\;\times \;\mathrm{Content}\;(\%)\;}\;\times \;100$$

(4) $$\mathrm{Released}\;\mathrm{Flavonoids}\;(\%)=\frac{\sum_{i=1}^{t}\mathrm{Ci}\;\times \;\mathrm{Vi}+\mathrm{Ct}\;\times \left(V-\sum \mathrm{Vi}\right)}{M\;\times \;\mathrm{Content}\;(\%)}\;\times \;100$$

where Ci is the measured concentration (mg GAE/L), Vi is the volume of the sample retrieved, V is the total volume of the medium (both volumes were constant), Ct is the total concentration of previous times (mg GAE/L or mg QE/L), M is the mass of particles used in the assay (mg), and Content (%) is the mass of extract in the phytosomes divided by the mass of encapsulated particles multiplied by 100.

#### 2.3.9. Statistical Analysis

All experiments were performed in triplicate (n = 3), and the data are expressed as mean ± SD. One or two-way ANOVA followed by Tukey’s test was used for statistical analysis of the results. All statistical analyses were performed using GraphPad Prism v. 10.0 (GraphPad software Inc., San Diego, CA, USA) and *p* < 0.05 was considered statistically significant.

## 3. Results

### 3.1. Phytochemical Profile of the Different Extracts of the Ice Plant

The Folin–Ciocalteu technique was applied in the different extracts of the ice plant, using gallic acid as a standard, and the concentration range was considered linear (R^2^ = 0.99). The results are presented in Table 1. Statistically significant differences were obtained among the different extracts studied (*p* < 0.0001) regarding the total polyphenolic content.

The extract that yielded the highest content in polyphenolic compounds was the one obtained from the solvent Ethanol:Acetone (80:20, *v*/*v*). It is worth noting that it is the only solvent without water present, which indicates that acetone could be an alternative to aqueous solvents. On the other hand, the extract that yielded the lowest polyphenolic content was the aqueous extract, followed by an ethanolic treatment, thus regarded as the less effective one.

The colorimetric aluminum chloride method was applied using quercetin as a standard, and the concentration range was considered linear (R^2^ = 0.99). The results are presented in Table 2. Statistically significant differences were obtained among the different extracts studied (*p* < 0.0001) regarding the total flavonoid content.

The Ethanol:Acetone 80:20 (*v*/*v*) extract stood out with the highest content of total flavonoids, followed by the extract obtained from the solvent Ethanol:Water 80:20 (*v*/*v*), with 761.7 ± 11.6 mg QE/100 g FW and 569.3 ± 17.0 mg QE/100 g FW, respectively. These results were consistent with the total polyphenolic content (TPC), as the extract obtained from the solvent Ethanol:Acetone 80:20 (*v*/*v*) was expected to present the highest content of flavonoids, suggesting this solvent again as the most effective for the polyphenolic extraction.

### 3.2. Physicochemical Characterization of Phytosomes

The phytosomal formulation was characterized via the DLS and the electrophoretic mobility assay methods, and the results are presented in Table 3.

An average particle size of 233.8 ± 2.0 nm was obtained, which is within the nanoscale range commonly reported for phytosomal formulations, as exemplified in the Discussion section. A reduced particle size, as obtained herein, increases the surface area-to-volume ratio and may facilitate the interaction with biological membranes, potentially contributing to improving the absorption rate, one of the objectives of the phytosomal formulations prepared in this work [41]. The PdI, an indicator of size homogeneity of the formulation particles, was lower than 0.3, indicating a relatively narrow size distribution of the extract-loaded phytosomes [32,35]. The zeta potential, a key indicator of colloidal dispersion physical stability against particle aggregation, is typically considered stable when |ζ| ≥ 30 mV [32,35]. Although the zeta potential value is slightly lower than this empirical threshold exclusively associated with electrostatic stabilization, it is within the moderate stability range, thus suggesting the presence of enough repulsion forces to avoid the aggregation of the particles [34].

### 3.3. Evaluation of EE

EE determination of the phytosomal formulation was performed by applying the Folin–Ciocalteu method. Considering that the total polyphenolic content of the phytosomal formulation supernatant was 1077 ± 4.2 mg GAE/L and that of the extract obtained from the solvent Ethanol:Acetone 80:20 (*v*/*v*) was 32,000 ± 56.6 mg GAE/L, an EE of 96.63% was obtained. This high EE value suggests a highly efficient complex formation between the polyphenolic constituents and phosphatidylcholine, supporting the suitability of the phytosomal approach for encapsulating bioactive compounds from the *Mesembryanthemum crystallinum* extract.

### 3.4. Quantification of Antioxidant Activity

The antioxidant activity was measured using the DPPH assay, which is sensitive even at low analyte concentrations [42]; the results are presented in Table 4. The extract with the highest antioxidant activity is the one obtained from the solvent Ethanol:Acetone 80:20 (71.2 ± 0.3%), and the lowest antioxidant activity was attributed to the aqueous extract (0.8 ± 0.3%). Statistically significant differences were obtained among the different formulations studied (*p* < 0.0001) regarding the antioxidant activity.

Comparing the activity to the quercetin (10 mg/mL) (positive control), the ethanolic extract with acetone is the only one that has relatively similar activity (ca. 81%, if considering the extract antioxidant activity/quercetin antioxidant activity). Consistently, this extract contains antioxidants able to reduce and stabilize DPPH and quercetin. These findings were consistent with the highest TPC and total flavonoid content (TFC), also reported for this extract.

Phytosomes presented an antioxidant activity of 16.0 ± 1.3%. This relatively low value was expected because the encapsulation of bioactive compounds may limit their immediate accessibility to free radicals in the assay medium. In other words, the antioxidants are encapsulated within the phytosomal structures and are therefore not readily available in their free form to exert radical scavenging activity. Additionally, dilution effects and the physicochemical nature of the vesicular system may also contribute to the reduced apparent activity measured in vitro.

### 3.5. In Vitro Release Studies

**Figure 2:**
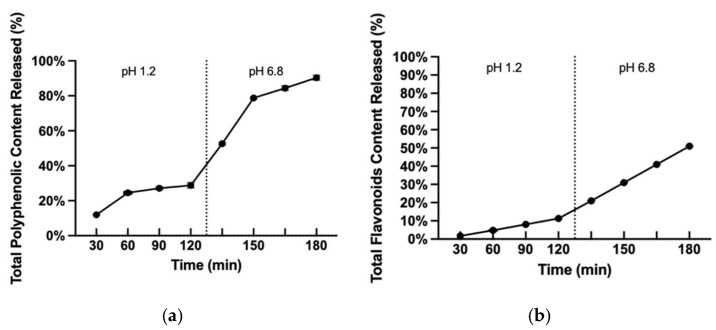
In vitro release profile of the TPC (%) (**a**) and of the TFC (%) (**b**) of the phytosomal formulation.

The in vitro release assay was performed through the determination of TPC (Figure 2a) and TFC (Figure 2b), thus verifying the polyphenolic release in a pH gradient mimicking the GIT. In the starting volume (1 mL), there was ca. 10 mg of the encapsulated extract. Approximately 90% of the TPC and 51% of the TFC release in the phosphate medium were achieved at the end of the experiment. The high TPC indicates the release of compounds into the medium since FC reacted with them. However, TPC reached only 90% and not 100% after 3 h, suggesting that not all polyphenolic content in the extract was released by the end of the experiment. This was further supported by the TFC only reaching 51% after 3 h.

**Figure 3:**
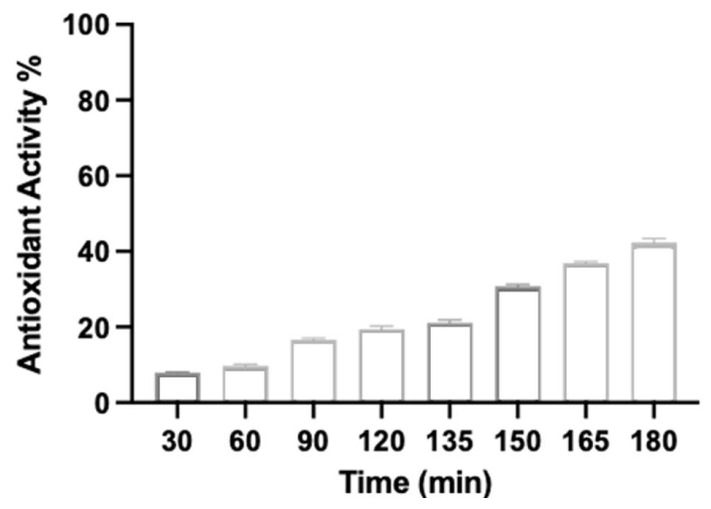
Antioxidant activity (%) over time in a pH gradient. Times 30–120 min correspond to pH 1.2, and times 135–180 min correspond to pH 6.8 (mean value ± SD).

The antioxidant activity (Figure 3) was also analyzed, and it is worth noting that the assay did not include gastric and intestinal enzymes. An increase in antioxidant activity was verified with time, reaching a maximum of 42% after 180 min in the phosphate medium. Although not a high percentage, it confirms the release of phytochemicals responsible for the antioxidant activity. This is in line with the TFC release, being justified by the availability of antioxidants in the medium throughout the experiment in comparison with the free extract.

## 4. Discussion

Some studies have reported the polyphenolic and flavonoid content of *Mesembryanthemum crystallinum* L., but different extraction solvents, conditions and methodologies were employed, which may contribute to variability in the reported values. The optimal solvent for extracting a significant amount of phenolic and flavonoid compounds among all the extracts was the Ethanol:Acetone 80:20 (*v*/*v*), being able to extract 3200.3 ± 56.7 mg GAE/100 g and 761.7 ± 11.6 mg QE/100 g of phenolic and flavonoid compounds, respectively.

Comparing the results obtained in this study with those of Calvo et al. [10], the total polyphenolic content found was lower than theirs. When extracted with an acidified mixture 50% ethanol *v*/*v*, Calvo and colleagues obtained 578.44 mg GAE/g in the ice plant. Different studies, such as Sousa et al. [23] and He et al. [43], reported that methanolic extracts (80%, *v*/*v*) of 1–2 g of the ice plant contained less than 120 µg GAE/g and 100–200 µg GAE/g, respectively. For He et al. [43], the range comes from the different biomasses of ice plants grown on several salt concentrations. Rincón-Cervera et al. [44] observed that the wild plant and the plant, cultivated at high light intensities and extracted with methanol 60% *v*/*v*, registered 3.4 and 2.6 mg GAE/g FW and TFC of 1.0 and 0.9 mg QE/g FW, respectively. Analyzing the influence of different combinations of red and blue LEDs on the ice plant growth, Kim et al. [45] observed that 1 g of the plant growth with increasing blue light ratio and extracted with methanol 80% *v*/*v*, presented 0.04 mg GAE/g FW, the highest concentration of the rest, despite having the smallest growth. The same tendency was observed for TFC, where the same plant registered 0.1 mg/g FW rutin equivalent (RE). Alshalmani and colleagues [5] reported ethanolic extracts with 414.7 mg/g tannic acid equivalent (TE) and 146.2 mg QE/g. Thus, methanolic extracts found in the literature reported lower TPC and TFC than those observed in this work. Additionally, Tuyen et al. [21] observed that ethanolic extracts (60:40, *v*/*v*) of the ice plant cultivated at different salt concentrations, presented 205.2 mg GAE/100 g FW and 32.0 mg QE/100 g FW for the highest salt concentration. The findings were lower than those observed in this study, compared with the studied ethanolic and with acetone extracts, but are higher than those obtained with Ethanol:Water 50:50 (*v*/*v*).

It is important to note that the extraction method chosen on each account influenced the yield of polyphenolic matter. In the extraction method, it is crucial to optimize time, temperature, solvent, and even the composition of the sample prior to the extraction to avoid the destruction of the polyphenolic fraction [42]. For example, the act of macerating the leaves, using a bath at a certain temperature or for a prolonged period in a chosen solvent, would improve the chances of the recovery of a rich extract at the end of the process [42]. Focusing on extracting solvents, they need to be generally non-toxic and be able to solubilize both polar and nonpolar phytocompounds from plant matrices [36,40]. Solvent polarity is directly proportional to the phytocompound’s solubility [41,42]. Thus, the higher the polarity, the greater the solubility of phytochemicals. This is due to a better diffusion of the solvent with the polyphenolic matrix, destabilizing the existing hydrogen bonds and leading to a higher solvation of the phytocompounds [40,42]. Polyphenols derivates are usually hydrophilic polar compounds, although this is dependent on the rings and their conjugations [43], so common extracting solvents are water, ethanol, acetone, and methanol [42]. Ethanol is a polar solvent, safe for human handling and the environment, and generally compatible with the reagents used for future assays [42]. Acetone is a polar organic aprotic solvent that is known for extracting polyphenols of higher molecular weight, whereas methanol is a polar aliphatic alcohol known for effectively extracting phytocompounds of smaller molecular weight. However, issues arise as it is more toxic for handling than ethanol [42]. Generally, ethanol is known for being able to extract flavonoids and associated glycosides and methanol, phenolic acids and catechins [44].

To maximize yield, it is common to combine two solvents to extract different polyphenols of different molecular weights and affinities. For instance, Do et al. [42] showed that high percentages of acetone and ethanol in water yield the highest TPC and TFC of *L. aromatica* extracts, whereas water alone yielded the least amount. The same was observed in this study, where ethanolic extracts and Ethanol:Acetone 80:20 *v*/*v* presented a better performance than their aqueous counterparts. This was especially noted on extracts obtained from Ethanol:Acetone 80:20 (*v*/*v*), indicating a better solubility of the compounds in acetone than in water. This can be explained due to the polar carbonyl group and non-polar methylated regions of the acetone [45]. Therefore, although water is more polar than acetone, certain compounds, such as hydroxycinnamic and hydroxybenzoic acids that exhibit non-polar characteristics due to the aromatic rings, would be associated better with acetone rather than water [36,46,47]. Ultimately, aspects such as the chemical structure and molecular weight, number, and position of hydroxyl groups dictate the solubility in solvents and therefore, the yield of extraction [48]. Moreover, differences in solvent-to-solid ratios may also influence extraction efficiency.

The formulation of the Ethanol: Acetone 80:20 *v*/*v* into phytosomes was carried out because of the phytochemical profile results. To the best of our knowledge, phytosomal formulations with *M. crystallinum* extract do not exist in the literature. Nonetheless, it is possible to compare the findings with already well-researched phytosomes. The physical characterization of the phytosomes formulated in this study was considered close to optimum, since size, zeta potential and PdI were fairly within the acceptable range [38]. The phytosomal formulation had a mean particle size of 233.8 ± 2.0 nm, a PdI of 0.20 ± 0.02, and a zeta potential of −27.3 ± 1.9 mV. Furthermore, the phytosomes demonstrated a high EE (96.63%). These results indicate that the current formulation can be further studied for oral therapeutic use. Similar physical characterization ranges were found by Portela et al. with phytosomes and seaweed extracts [49]. It was reported that the seaweed phytosomes’ size ranged from 118 to 366 nm, with the size increasing with each additional layer to the nanocarrier. The PdI parameter included results from 0.7 to 0.3, which were slightly above the ideal <0.3, with the higher records attributed to the larger-sized particles [49]. The zeta potential of the seaweed phytosomes did not align with the ideal range of ±30 mV, being justified by the lack of PEG layers, which could help in decreasing the zeta potential [49]. Deleanu and colleagues [50], when formulating ginger rhizomes and rosehip phytosomes with a 46.3 mg GAE/100 g, found that the nanoparticles with different ratios of phosphatidylcholine and extract presented an average size from 190 to 780 nm. PdI and zeta potential did not reach an adequate range, but the zeta potential reached −22 mV. The PdI values of 0.5 were due to high concentrations of extract, which can increase the particle size and give the system size heterogeneity [50]. Considering the steric stabilization conferred by the phospholipids, the charge contribution of the complexed polyphenols, and the maintenance of size and PdI throughout storage, the formulation presented adequate colloidal stability for the period studied.

Direito et al. [38], the reference source for phytosomal preparation, also reported a high encapsulation entrapment of phytosomes loaded with *Diospyros kaki* extract (93.6% through the FC assay). Deleanu et al. [50] reported EE percentages above 85% with the ratio 1:1 (extract ginger rhizomes alone: PC). One of the highest (91.3%) was obtained when compared to other ratios, also including extract rosehips, showing that ginger rhizomes are better encapsulated when alone. It is worth noting that when trying to combine two extracts, the ratio 0.5:0.5:1 (extract ginger rhizomes:extract rosehips:PC) showed a better performance (83.3% and 94.3% for each extract, respectively) than the ratios of 0.75:0.25:1 and 0.9:0.1:1. Deleanu et al. [50] formulated phytosomes at different ratios and emphasizes the importance of ratio optimization of phosphatidylcholine and extract available as well as the effectiveness of the chemical bonds between the phospholipid and the phytochemicals in the phytosomal formulations [44,51].

The phytosomal formulation of this study reported a high EE (96.63%), which confirms that it carries the phytochemicals with therapeutic potential from *M. crystallinum*. This high degree of entrapment is particularly significant because polyphenols are often unstable and poorly absorbed in their free form. By encapsulating them within phytosomes, their stability and solubility in biological membranes are greatly improved, which in turn enhances their potential bioavailability.

Regarding the antioxidant activity of the ice plant, the results were in line with those found in the literature, confirming that the plant exhibited significant antioxidant activity (reaching 71.2%). It is worth noting that the present study did not go beyond the DPPH assay, as it is still a preliminary study. However, for future research, a wide variety of studies, such as ferric-reducing–antioxidant power (FRAP) assay, should be incorporated to better illustrate the antioxidant activity of the plant [42].

The in vitro release suggested that certain antioxidant activity was retained (reaching a maximum of 42%), accompanied by 51% release of the flavonoid content at the end of the 3 h assay. The antioxidant activity reported by Tuyen et al. [21] was 75.7% for the ice plant grown on the highest salt concentration and extracted with 60% ethanol. For increasing concentrations of methanolic extracts, Alshalmani and colleagues [5] observed that the antioxidant activity reached 65.6%. The antioxidant activity reported in these studies is within the range observed in this work with the ethanolic extracts, mainly Ethanol:Acetone 80:20 *v*/*v* and Ethanol:Water 80:20 *v*/*v*. These findings support our results and highlight that the reduced antioxidant activity measured in vitro immediately after formulation does not indicate inefficacy, but rather successful encapsulation. By retaining antioxidants within the phytosomes, the system improves stability and ensures a gradual release, which is expected to enhance bioavailability and therapeutic effectiveness under physiological conditions. The in vitro release assay is important because bioaccessibility is directly linked to bioavailability [50]. Bioaccessibility refers to the number of compounds present after digestion in the gastrointestinal tract, which will then convert to compounds available to exert their bioactivities [50].

The maximum content released in the phosphate medium was achieved with a pH of 6.8 in all experiments, suggesting a tendency toward increased release at near-neutral pH under the experimental conditions. In neither release assay was a burst release noted, possibly attributed to the pH sensitivities of the formulation, making it slightly more resistant to acidic environments. Furthermore, it is important to note that the timeframe and conditions of the experiment represent a preliminary assessment of the release kinetics, given the gastric residence time, which may extend up to several hours depending on physiological conditions [52]. Therefore, to study the potential of the phytosomes in an oral formulation, future studies should include extended-release assays to better evaluate the time-dependent release profile. The incorporation of biorelevant media, such as simulated gastric and intestinal fluids containing digestive enzymes, would further improve physiological relevance [50]. Additionally, mathematical modeling of release kinetics using models such as Higuchi, or zero- and first-order kinetics is recommended to elucidate the underlying release mechanisms [32]. The phytosomal formulation included phosphatidylcholine, an amphipathic phospholipid that facilitates interaction with biological membranes, which can interact in a versatile manner in different environments and cells and is known for protecting encapsulated compounds in the gastrointestinal tract [53]. It does so through its chemical structure composed of a zwitterionic choline–phosphate polar head group and two hydrophobic fatty acid chains [53]. The structure provides a neutral surface, and it is predicted to form bilayers tightly packed, potentially reducing interactions with digestive enzymes and the acidic medium [53]. Barani et al. [32] highlighted that phytosomes, by forming stable complexes with phospholipids, not only improve membrane permeability but also contribute to controlled and sustained release profiles. Notably, the literature review indicated that phytosomes can exhibit pH-dependent behavior, being more resistant in acidic environments, while facilitating the gradual release of phytocompounds at near-neutral pH [32]. This mechanistic evidence supports the notion that the observed absence of burst release and preferential release at pH 6.8 in our study are characteristic features of phytosomal systems, which enhance stability in the gastrointestinal tract and increase the likelihood of improved therapeutic efficacy in vivo.

Phytosomes with extracts of ginger rhizomes and rosehip, in an environment that mimicked the gastrointestinal tract with the addition of digestive enzymes, showed better bioaccessibility (reaching almost 30%, 4 times more than the extracts alone) [50]. This shows the ability of phytosomes to withstand harsh conditions of the gastrointestinal tract and to be available post-gastrointestinal digestion to exert their bioactivities [50]. A study on phytosomes formulated from *Nicotiana tabacum* var. Virginia leaf extract showed that when increasing concentrations of the fresh leaf extracts, the nanoparticles presented an antioxidant activity similar to that of quercetin and gallic acid (exceeding 80%) [54]. Ortega-Pérez et al. [55] reported that although phytosomes with *Callistemon citrinus* leaf extract exhibited an antioxidant activity lower than the free extract, there were no statistically significant differences.

The antioxidant activity of phytochemicals is also associated with the modulation of endogenous antioxidant enzymes and radical scavenging capacity [10]. In vivo antioxidant studies done by Deleanu et al. [50] showed an increase in the expression of the SOD enzyme in mice enterocytes, with a peak of 91% at 10 µM for the phytosomes with the 0.5:0.5:1 ratio, which shows that the formulations can maintain the antioxidant activity of the phytocompounds. Importantly, the formulations prevented the degradation of the active compounds in the stomach, suggesting that phytosomes provide protection in acidic environments and promote controlled release under near-neutral environments. Likewise, Direito and colleagues [38] compared the percentage of decay of antioxidant activity of free extracts and phytosomes loaded with *D. kaki* throughout 6 months of storage at three different temperatures (4 °C, 25 °C and 40 °C), and it was observed that phytosomal formulations protected the encapsulated extract from degradation and maintained its antioxidant activity better than the free extract. Similarly, when encapsulating *Hylocereus costaricensis* phenolic extract into phytosomes, Direito et al. [56] demonstrated comparable antioxidant activity of the phytosomes (38.3%) with respect to the free extract (33.4%), with nearly half of the phenolic content. These findings suggest that the encapsulation of extracts enables an efficient release of phytocompounds, while conserving functionality at reduce dosages [56]. These studies demonstrated the protective and antioxidant ability of phytosomes. The results of this work showed that although the antioxidant activity post-release assay was reduced due to the quantity of TFC released into the medium, some activity was retained when compared to the free extract. Therefore, there is still a need to further investigate the activity of phytosomes by improved release assays with the inclusion of digestive enzymes, to better draw conclusions from. There is room for the development of an oral formulation of phytosomes containing the ice plant extract. If the formulation aims for commercialization, steps required for the approval include a proper phytochemical and target identification, formulation optimization, and preclinical and clinical testing [57]. Possible challenges that could be found in the preclinical and clinical landscape are regulatory hurdles, including the case of borderline products, where further analysis is needed to determine if the product could be a drug or a supplement [57]. Another issue is the lack of established guidelines, especially for nanoparticles containing phytochemicals, to ensure safety. Additional challenges will possibly reside in the standardization of ice plant extracts, batch-to-batch variability and patient heterogeneity [58,59].

This present study demonstrated that the extracts from *Mesembryanthemum crystallinum* possess substantial antioxidant activity, and the formulation of phytosomes loading this extract presents a promising approach, which is expected to improve its pharmacokinetics and bioavailability. Considering the bioactivity and the potential reported for the ice plant in previous studies, as well as in this work, future research should continue to focus on further optimizing and characterizing phytosomal delivery systems with the ice plant extract.

## 5. Conclusions

*M. crystallinum* L. is a halophyte plant that possesses a rich composition of bioactive compounds, particularly phenols and flavonoids, which contribute to its strong antioxidant potential. The present study demonstrates that appropriate extraction strategies can maximize the recovery of these compounds and highlight the promise of the plant as a natural source of antioxidants. These findings also reinforce the potential relevance of *M. crystallinum* in the prevention or management of oxidative stress-related conditions.

Considering the well-recognized limitations of phytochemicals, namely their low bioavailability, the study also explored phytosomes as a novel drug delivery system for the extract. The phytosomal formulation showed favorable physicochemical characteristics and high EE, indicating the successful incorporation of the bioactives. Although the in vitro release assays revealed lower antioxidant activity compared to the free extract, the results still confirmed the ability of phytosomes to gradually release phytochemicals under physiological conditions. This sustained release profile suggests that phytosomes could enhance the stability and potential bioactivity of *M. crystallinum* extracts.

Altogether, the study presents a preliminary yet significant contribution by proposing *M. crystallinum*-loaded phytosomes as a promising candidate for future therapeutic applications. To fully validate this approach, further studies including enzymatic digestion models and in vivo evaluation are required to confirm the translational relevance. Such studies would not only consolidate the bioactivity prospects of *M. crystallinum* in different pathologies but also expand the applicability of phytosome-based delivery systems for halophyte-derived bioactives more broadly.

## Figures and Tables

**Table 1:** Total polyphenolic content (mg GAE/100 g FW) of different solvent extracts of the ice plant (mean value ± SD). The values obtained show statistically significant differences in the total polyphenolic levels present in the different extracts (*p* < 0.0001).

Ice Plant Extracts	Total Polyphenolic Content (mg GAE/100 g FW)
Ethanol: Water 80:20 (*v*/*v*)	2384.9 ± 164.5
Ethanol: Water 50:50 (*v*/*v*)	1666.9 ± 35.2
Ethanol: Acetone 80:20 (*v*/*v*)	3200.3 ± 56.7
Water	966.9 ± 20.4
Water Treated with Ethanol	392.5 ± 25.5

**Table 2:** Total flavonoid content (mg QE/100 g FW) of different solvent extracts of the ice plant (mean value ± SD). The values obtained show statistically significant differences in the total flavonoids levels present in the different extracts (*p* < 0.0001).

Ice Plant Extracts	Total Flavonoid Content (mg QE/100 g FW)
Ethanol: Water 80:20 (*v*/*v*)	569.3 ± 17.0
Ethanol: Water 50:50 (*v*/*v*)	120.7 ± 2.3
Ethanol: Acetone 80:20 (*v*/*v*)	761.7 ± 11.6
Water	267.7 ± 14.4
Water Treated with Ethanol	4.7 ± 2.1

**Table 3:** Physicochemical characterization of phytosomal formulation loaded with ice plant extract (mean value ± SD).

	Loaded Phytosomes
Average Particle Size (nm)	233.8 ± 2.0
Polydispersity Index (PdI)	0.2 ± 0.02
Zeta Potential (mV)	−27.3 ± 1.9

**Table 4:** Antioxidant activity (%) of quercetin, extracts and loaded phytosomes (mean value ± SD). The antioxidant activities registered are significantly different from each other (*p* < 0.0001).

	Antioxidant Activity (%)
Quercetin (positive control)	88.0 ± 0.2
Ethanol: Water 80:20 (*v*/*v*)	59.7 ± 0.2
Ethanol: Water 50:50 (*v*/*v*)	27.8 ± 0.3
Ethanol: Acetone 80:20 (*v*/*v*)	71.2 ± 0.3
Water	0.8 ± 0.3
Water Treated with Ethanol	3.7 ± 0.1
Loaded phytosomes	16.0 ± 1.3

## Data Availability

The original contributions presented in this study are included in the article. Further inquiries can be directed to the corresponding authors.

## Abbreviations

The following abbreviations are used in this manuscript:

DPPH	2,2-diphenyl-1-picrylhydrazyl
EE	Encapsulation Efficiency
FC	Folin–Ciocalteu
PC	Phosphatidylcholine
PdI	Polydispersity Index
TFC	Total Flavonoid Content
TPC	Total Polyphenolic Content
RE	Rutin Equivalent
TE	Tannic Acid Equivalent
